# Changes in dead space components during pressure-controlled inverse ratio ventilation: A secondary analysis of a randomized trial

**DOI:** 10.1371/journal.pone.0258504

**Published:** 2021-10-13

**Authors:** Go Hirabayashi, Yuuki Yokose, Kohei Nagata, Hiroyuki Oshika, Minami Saito, Yuki Akihisa, Koichi Maruyama, Tomio Andoh

**Affiliations:** Department of Anaesthesiology, Mizonokuchi Hospital Teikyo University School of Medicine, Kanagawa, Japan; Srebrnjak Children’s Hospital, CROATIA

## Abstract

**Background:**

We previously reported that there were no differences between the lung-protective actions of pressure-controlled inverse ratio ventilation and volume control ventilation based on the changes in serum cytokine levels. Dead space represents a ventilation-perfusion mismatch, and can enable us to understand the heterogeneity and elapsed time changes in ventilation-perfusion mismatch.

**Methods:**

This study was a secondary analysis of a randomized controlled trial of patients who underwent robot-assisted laparoscopic radical prostatectomy. The inspiratory to expiratory ratio was adjusted individually by observing the expiratory flow-time wave in the pressure-controlled inverse ratio ventilation group (n = 14) and was set to 1:2 in the volume-control ventilation group (n = 13). Using volumetric capnography, the physiological dead space was divided into three dead space components: airway, alveolar, and shunt dead space. The influence of pressure-controlled inverse ratio ventilation and time factor on the changes in each dead space component rate was analyzed using the Mann-Whitney U test and Wilcoxon’s signed rank test.

**Results:**

The physiological dead space and shunt dead space rate were decreased in the pressure-controlled inverse ratio ventilation group compared with those in the volume control ventilation group (p < 0.001 and p = 0.003, respectively), and both dead space rates increased with time in both groups. The airway dead space rate increased with time, but the difference between the groups was not significant. There were no significant changes in the alveolar dead space rate.

**Conclusions:**

Pressure-controlled inverse ratio ventilation reduced the physiological dead space rate, suggesting an improvement in the total ventilation/perfusion mismatch due to improved inflation of the alveoli affected by heterogeneous expansion disorder without hyperinflation of the normal alveoli. However, the shunt dead space rate increased with time, suggesting that atelectasis developed with time in both groups.

## Introduction

Previously, we studied the changes in dead space components within a short duration (30 min) of each ventilator mode using a cross-over study design, and reported that pressure-controlled inverse ratio ventilation (PC-IRV) reduces the physiological dead space (VD_phys_) [1]. We believe that PC-IRV might be a lung-protective ventilation strategy. However, another randomized controlled study reported no differences between the lung-protective properties of PC-IRV and volume-control ventilation (VCV) when performed for >2 h in robot-assisted laparoscopic radical prostatectomy, as determined by the changes in serum cytokine levels [2]. However, serum cytokine levels are affected not only by the ventilator setting, but also by duration, surgical invasion, bleeding, and any stress to the patient.

VD_phys_ represents the overall ventilatory efficiency, including circulatory dynamics, and a total of ventilation/perfusion (${\overset{\cdot }{V}}_{A}/\overset{\cdot }{Q}$) mismatch. PC-IRV or pressure-controlled ventilation with sufficient inspiratory time might reduce the VD_phys_ [1–7]. Using volumetric capnography, the VD_phys_ is divided into three dead space components: airway dead space (VD_aw_), alveolar dead space (VD_alv_), and shunt dead space (VD_shunt_) [1]. Each dead space component represents a respective ${\overset{\cdot }{V}}_{A}/\overset{\cdot }{Q}$ mismatch, enabling us to understand the heterogeneity and elapsed time changes of ${\overset{\cdot }{V}}_{A}/\overset{\cdot }{Q}$ mismatch, which would contribute to practicing the open-lung approach for ventilation.

We studied the changes in dead space components induced by PC-IRV and the elapsed time and aimed to evaluate the ventilation-perfusion characteristics of PC-IRV and VCV modes and their sustainability. We hypothesized that PC-IRV reduces dead space and improves ${\overset{\cdot }{V}}_{A}/\overset{\cdot }{Q}$ mismatch, and that PC-IRV may be an alternative to the open-lung approach for ventilation.

## Materials and methods

### Study design and ethics

This study was a secondary analysis of a previously published single-center, prospective, single-blinded, randomized controlled trial [2]. Ethical approval for this randomized controlled trial (No. 17–063) was obtained from the Ethical Committee of Teikyo University School of Medicine, Tokyo, Japan (Chairperson and Dean: H. Takigawa) on 12 September 2017, and the trial was registered with the University Hospital Medical Information Network Clinical Trials Registry (UMIN000029552). Patients aged 18–85 years with American Society of Anesthesiologists (ASA) physical status I or II and scheduled for robot-assisted laparoscopic radical prostatectomy were included in this study. The exclusion criteria were ASA physical status 3–5, and/or a history of pneumothorax and lung surgery. Written informed consent was obtained from all qualifying patients. Researchers at the Teikyo Academic Research Centre randomized the patients to the VCV (n = 14) or PC-IRV groups (n = 14) with a 1:1 allocation ratio using the envelope method, after generating the allocation sequence. Only the patients remained blinded during the whole study procedure. The study was conducted in the Mizonokuchi Hospital Teikyo University School of Medicine, Kanagawa, Japan, between December 2017 and September 2018. The original Japanese study protocol approved by the Ethical Committee of Teikyo University School of Medicine is shown in S1 File, and English protocol in S2 File. This study adhered to the CONSORT guidelines, and the CONSORT checklist is provided in the S3 File.

### Anesthesia protocol

Routine patient monitoring included electrocardiography, pulse oximetry, non-invasive arterial blood pressure measurement, and anesthetic gas CO_2_ analysis. The Vigileo with the Flo-Trac sensor (Edwards Lifesciences, Irvine, CA, USA) was used to monitor continuous radial arterial pressure, cardiac index, and stroke volume variation. Mainstream CO_2_ and flow sensors were attached to the proximal end of the tracheal tube to enable volumetric capnography (Senko Medical Instrument Co. Ltd., Tokyo, Japan). Anesthesia was induced by administering 1–3 mg.kg^-1^ intravenous propofol and 2–4 μg.kg^-1^ fentanyl. Tracheal intubation was performed with an 8.0-mm cuffed tube, following administration of 0.6–0.9 mg.kg^-1^ rocuronium. Anesthesia was maintained with volatile anesthetic gas composed of 3–4% desflurane, supplemented with continuous intravenous infusions of 0.2–0.3 μg.kg^-1^.min^-1^ remifentanil and intermittent intravenous injections of 0.1–0.2 mg.kg^-1^ rocuronium or 1–2 μg.kg^-1^ fentanyl when needed.

An anesthesia ventilator (Avance Carestation, Datex-Ohmeda, GE Healthcare, Helsinki, Finland) was used. The initial ventilator settings included the volume-control mode, tidal volume set at 8–10 ml.kg^-1^ ideal body weight (IBW) (i.e. 50 + 0.91 × [height in cm—152.4]), respiratory rate of 12 breaths.min^-1^, baseline airway pressure (BAP; used as setting positive-end expiratory pressure) of 5 cmH_2_O, 0.5 fraction of inspired oxygen (FiO_2_), and an inspiration to expiration (I:E) ratio of 1:2. A pause ratio of 20% was set to measure the plateau pressure. The ventilator settings were switched to the PC-IRV or VCV strategy following the establishment of the 25–30° Trendelenburg position and CO_2_ pneumoperitoneum at 12 mmHg.

### Interventions and ventilatory strategies

The PC-IRV strategy included the pressure-control ventilation-volume guarantee mode, in which the airway pressure was adjusted to achieve a target tidal volume with plateau pressures permitted to rise to an upper limit of 30 cmH_2_O. BAP was switched off. I:E ratios of 2:1 or 1.5:1 were selected so that inspiration started at the midpoint between the expiratory flow change point and the return point to the expected baseline to avoid hyperinflation. The VCV strategy included the volume-control mode with an I:E ratio of 1:2. A pause ratio of 20% was set to measure the plateau pressure. A target tidal volume was set at <10 ml.kg^-1^ IBW to prevent the plateau pressure from exceeding the upper limit of 30 cmH_2_O. BAP was set to 5 cmH_2_O. In both strategies, the initial F_i_O_2_ was 0.5 and the initial respiratory rate was 12 breaths.min^-1^. This allowed an increase in the respiratory rate to an upper limit of 18 breaths.min^-1^ to achieve an arterial partial pressure of CO_2_ (P_a_CO_2_) of <50 mmHg, which was estimated from the end-tidal CO_2_ (E_T_CO_2_) changes and the differences between E_T_CO_2_ and P_a_CO_2_ on arterial blood gas analysis. Hypercapnia (>50 mmHg) was permitted if the respiratory rate increased to 18 beats.min^-1^ with a plateau pressure of 30 cmH_2_O.

Hemodynamics were maintained with a mean arterial pressure >70 mmHg, a cardiac index >2 L.min^-1^.m^-2^, and a stroke volume variation <15%. If the mean arterial pressure fell below 70 mmHg, intravenous ephedrine (4–8 mg) was administered. If the stroke volume variation exceeded 15%, an additional intravenous fluid challenge was provided with 10 ml.kg^-1^ of Ringer’s acetate solution or hydroxyethyl starch. Pulse oximetry-monitored oxygen saturation was allowed to drop to a lower limit of 93%. When these parameters exceeded the predetermined limits, the ventilator setting was changed by increasing the respiratory rate and F_i_O_2_, and increasing or decreasing the set tidal volume.

### Outcomes and novel theory of volumetric capnography

**Fig 1:**
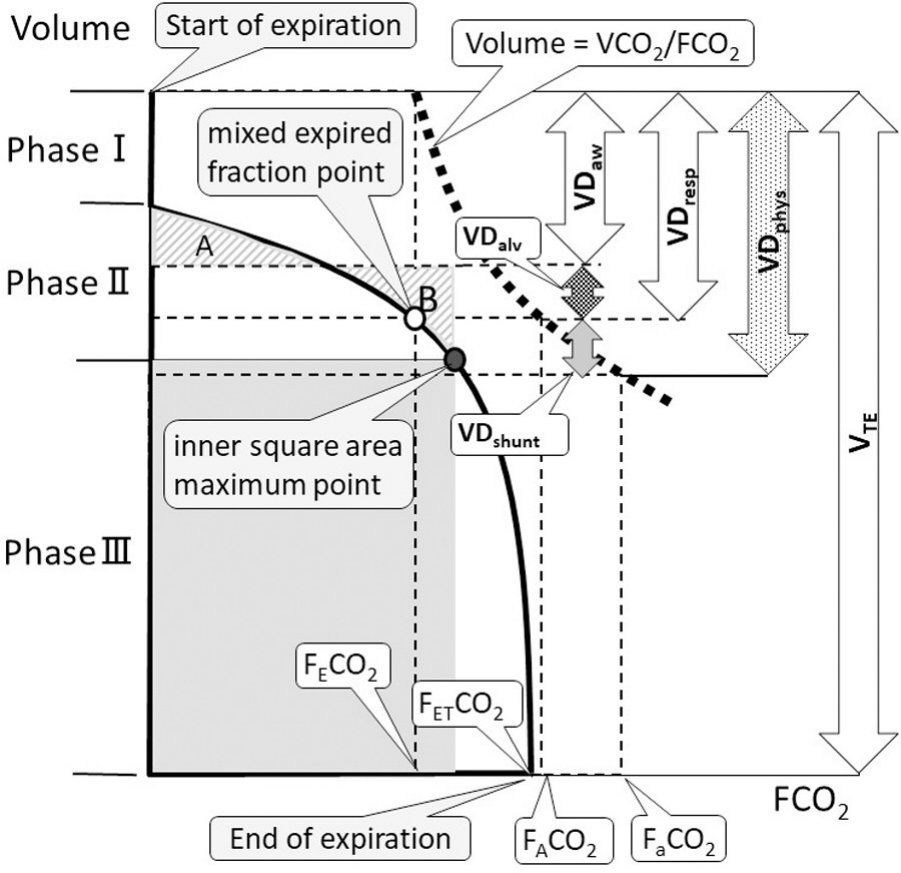
Components of dead space and volumetric capnography. The expired tidal volume on the Y axis is plotted against the partial pressure of expired CO_2_ on the X axis in order to express the curve of ‘Volume = VCO_2_/FCO_2_’. Phase I represents CO_2_-free and pure dead space, phase II represents the transition between airway and alveolar gas, and phase III represents alveolar gas. Phase II ends at the inner square area maximum point. The VD_aw_ is determined by applying Fowler’s equal area method (area A equals to B). The volume from the start of expiration to the partial pressure of mixed expired CO_2_ point on the Y axis is defined as respiratory dead space (VD_resp_). Thus, alveolar dead space (VD_alv_) is calculated as VD_resp_−VD_aw_, shunt dead space (VD_shunt_) is calculated as VD_phys_−VD_resp_. Abbreviations: VD_phys_, physiological dead space; VD_aw_, airway dead space; VD_alv_, alveolar dead space; VD_shunt_, shunt dead space; V_TE_, expired tidal volume; VCO_2_, expired tidal volume of CO_2_; FCO_2_, fractional concentration of CO_2_ [FCO_2_ = PCO_2_ (PB–PH_2_O)^-1^ = PCO_2_ (760–47)^-1^; PB, barometric pressure; PH_2_O, water vapor pressure at 37°C]; P_E_CO_2_, mixed expired partial pressure of CO_2_; P_ET_CO_2_, end-tidal partial pressure of CO_2_; P_A_CO_2_, alveolar partial pressure of CO_2_; and PaCO_2_, arterial partial pressure of CO_2_ are expressed as FCO_2_ in this figure.

The primary outcome included the physiological dead space rate (VD_phys_/V_TE_) (V_TE_; expired tidal volume), calculated as (P_a_CO_2_—P_E_CO_2_)/P_a_CO_2_ (P_E_CO_2_; mixed expired partial pressure of CO_2_), which represented the overall ventilatory efficiency including circulatory dynamics and a total of ${\overset{\cdot }{V}}_{A}/\overset{\cdot }{Q}$ mismatch. Using the novel theory of volumetric capnography (S4 File), the VD_phys_ was divided into VD_aw_, VD_alv_, and VD_shunt_; (VD_phys_ = VD_aw_ + VD_alv_ + VD_shunt_) (Fig 1).

VD_aw_ was analyzed non-invasively and geometrically using Fowler’s equal area method [8]. VD_aw_ is a functional evaluation of the airway space volume, representing extra-alveolar ${\overset{\cdot }{V}}_{A}/\overset{\cdot }{Q}$ = ∞ mismatch [1, 9–11]. The volume from the start of expiration to the mixed expired CO_2_ fraction point on the Y axis was defined as respiratory dead space (VD_resp_). VD_resp_ is a functional evaluation of the difference in CO_2_ partial pressure between alveolar and mixed expired gas, representing ${\overset{\cdot }{V}}_{A}/\overset{\cdot }{Q}$ >1 mismatch including extra-alveolar ${\overset{\cdot }{V}}_{A}/\overset{\cdot }{Q}$ = ∞ mismatch. VD_alv_ was defined as VD_resp_—VD_aw_. The VD_alv_ is a functional evaluation of the relative hyperinflation in the alveolar units, representing intra-alveolar ${\overset{\cdot }{V}}_{A}/\overset{\cdot }{Q}$ >1 mismatch. VD_shunt_ was calculated as VD_phys_−VD_resp_ or VD_phys_—VD_aw_—VD_alv_, which represented functional evaluation of relative hyper-perfusion and the difference in CO_2_ partial pressure between pulmonary artery and mixed expired gas, and intra and extra-alveolar ${\overset{\cdot }{V}}_{A}/\overset{\cdot }{Q}$ <1 mismatch. VD_shunt_ is a new definition and VD_alv_ was calculated as VD_resp_—VD_aw_ in this study, which was different from VD_alv_ calculated as ‘VD_phys_—VD_aw_‘ by Fletcher and colleagues [12].

The VD_aw_, VD_alv_, V_TE_, and P_E_CO_2_ were measured using volumetric capnography, and P_a_CO_2_ was measured using arterial blood samples. Each dead space rate was measured or calculated at T_Baseline_ (initial setting), T_20min_ (20 min after intervention), and T_2h_ (2 h after intervention).

Furthermore, the influence of PC-IRV (dead space changes depending on plateau time) and time factors (dead space changes with time, non-dependent on plateau time) on changes in each dead space component rate were analyzed. For the PC-IRV factor, sufficient plateau time or inspiratory time enhances gas diffusion between the airway and alveolar units and induces changes in the VD_aw_, which represented extra-alveolar ${\overset{\cdot }{V}}_{A}/\overset{\cdot }{Q}$ = ∞ mismatch caused by gas diffusion. PC-IRV may also induce changes in the VD_alv_/V_TE_ by causing intra-alveolar ${\overset{\cdot }{V}}_{A}/\overset{\cdot }{Q}$ >1 (excluding intra-alveolar ${\overset{\cdot }{V}}_{A}/\overset{\cdot }{Q}$ = ∞) mismatch, or relative hyperinflation due to changes in the plateau time or pressure, or hemodynamics. PC-IRV may induce changes in the VD_shunt_/V_TE_ by causing an intra-alveolar ${\overset{\cdot }{V}}_{A}/\overset{\cdot }{Q}$ <1 mismatch because sufficient plateau time contributes to the expansion of alveoli affected by heterogeneous expansion disorders, thereby enhancing gas diffusion from the pulmonary artery to the alveoli.

The time factor may induce changes in the VD_aw_/V_TE_ by changing the airway space volume and in the VD_alv_/V_TE_ by causing intra-alveolar ${\overset{\cdot }{V}}_{A}/\overset{\cdot }{Q}$ = ∞ mismatch; such as with pulmonary infarction for example. The time factor may induce changes in the VD_shunt_/V_TE_ by causing intra-alveolar ${\overset{\cdot }{V}}_{A}/\overset{\cdot }{Q}$ = 0 mismatch due to the development of atelectasis, or extra-alveolar ${\overset{\cdot }{V}}_{A}/\overset{\cdot }{Q}$ = 0 mismatch, such as an extra-alveolar shunt. In this study, there was a small effect of the extra-alveolar shunt in all cases; thus, changes in the VD_shunt_/V_TE_ caused by the time factor mainly indicate the development of atelectasis (Table 1).

**Table 1:** Interpretation of dead space components.

Dead space component		Affecting factor	${\overset{\cdot }{V}}_{A}/\overset{\cdot }{Q}$ mismatch
Physiological dead space (VD_phys_) **[Total of all** ${\overset{\cdot }{V}}_{A}/\overset{\cdot }{Q}$ **mismatch]**	**Airway dead space (VD**_**aw**_ **)**	**Time**	**Extra-alveolar ** ${\overset{\cdot }{V}}_{A}/\overset{\cdot }{Q}$ ** = ∞**
			Airway space volume change
		Dead space changes with time, non-dependent on plateau time	
	Functional evaluation of the airway space volume		
		**PC-IRV**	**Extra-alveolar ** ${\overset{\cdot }{V}}_{A}/\overset{\cdot }{Q}$ ** = ∞**
		Dead space changes depending on plateau time	Diffusion efficiency between alveolar units and airway
	**Alveolar dead space (VD**_**alv**_ **)**	**Time**	**Intra-alveolar ** ${\overset{\cdot }{V}}_{A}/\overset{\cdot }{Q}$ ** = ∞**
			Pulmonary infarction
	Functional evaluation of the relative hyperinflation in the alveolar units		
		**PC-IRV**	**Intra-alveolar ** ${\overset{\cdot }{V}}_{A}/\overset{\cdot }{Q}$ ** > 1**
			Relative hyperinflation, affected by plateau time, plateau pressure, and hemodynamics
	**Shunt dead space (VD**_**shunt**_ **)**	**PC-IRV**	**Intra-alveolar ** ${\overset{\cdot }{V}}_{A}/\overset{\cdot }{Q}$ ** < 1**
			Relative hyper-perfusion due to slow-opening alveoli that is improved by sufficient plateau time
	Functional evaluation of the relative hyper-perfusion in the alveolar or extra-alveolar units		
		**Time**	**Intra-alveolar ** ${\overset{\cdot }{V}}_{A}/\overset{\cdot }{Q}$ ** = 0**
			Atelectasis
			**Extra-alveolar ** ${\overset{\cdot }{V}}_{A}/\overset{\cdot }{Q}$ ** = 0**
			Extra pulmonary shunt

Abbreviations: PC-IRV, pressure-controlled inverse ratio ventilation; ${\overset{\cdot }{V}}_{A}/\overset{\cdot }{Q}$, ratio of ventilation to perfusion.

### Statistical analysis

The sample size was calculated as 11 participants per group to detect the differences in the VD_phys_/V_TE_, with a power of 80% and a type I error rate of 0.05, based on an estimated difference of 0.8 between the parameter’s estimated standard deviation (SD). Thus, a sample size of 13 or 14 participants per group in the primary study was sufficient for this investigation. Data are presented as median (IQR [range]). Comparisons between the groups were performed using the Mann−Whitney U test, and comparisons according to the measurement point using Wilcoxon’s signed rank test. The association between each dead space component rate and respiratory variables was evaluated using Spearman’s rank correlation.

P-values <0.05 were considered statistically significant. Statistical analyses were performed using R© version 3.5.2 (2018-12-20; 2018 The R Foundation for Statistical Computing).

## Results

**Fig 2:**
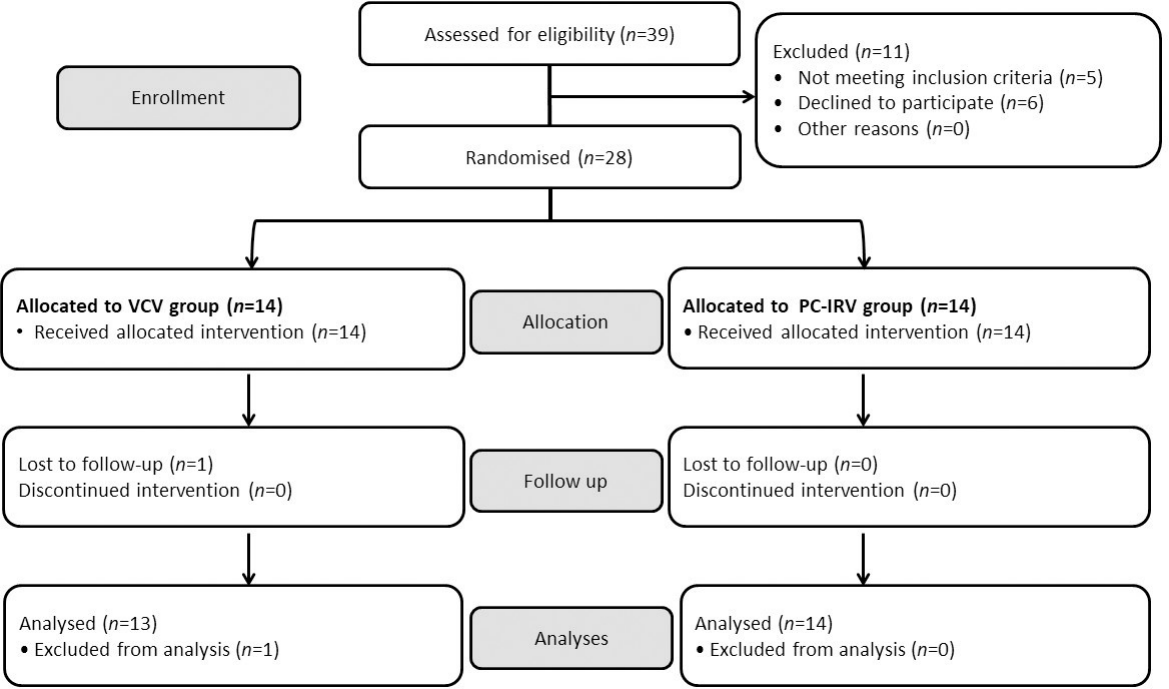
CONSORT diagram of patient recruitment for the comparison of volume-controlled ventilation and pressure-controlled inverse ratio ventilation.

Patient enrolment started on 6 December 2017. Thirty-nine consecutive patients undergoing robot-assisted laparoscopic radical prostatectomy were screened; five patients did not meet the inclusion criteria, and six refused to give consent. One patient in the VCV group was lost to follow-up due to a mistake in installing the volumetric capnography sensor. Thus, 13 patients from the VCV group and 14 from the PC-IRV group were analyzed (Fig 2). Dataset is provided in the S5 File.

There were no significant differences in the patient and surgical characteristics. No significant differences were observed in the respiratory or hemodynamic parameters, or the rate of each dead space component between the groups at T_Baseline_ with the same initial ventilator settings, including the VCV mode, in both groups (Table 2).

**Table 2:** Patient and surgical characteristics, and the respiratory and hemodynamic parameters at T_Baseline_ (20 min after the initial setting).

Variable	VCV group (n = 13)	PC-IRV group (n = 14)	p value
**Age; years**	68 (62–72 [58–77])	69.5 (68–72 [58–76])	0.643
**Height; cm**	168 (164–172 [163–176])	166 (162–172 [158–175])	0.307
**Weight; kg**	68 (63.6–72 [55–97])	70.7 (64.3–76.8 [52–103])	0.61
**Body mass index; kg.m** ^**-2**^	24 (23.9–25.8 [17.9–31.3])	25.6 (22.4–27.5 [20.8–33.6])	0.56
**FVC % predicted; %**	108 (93–117 [79–132])	109 (101–116 [80–134])	0.808
**FEV**_**1**_ **% predicted; %**	78 (73–83.7 [62–91])	81 (76.5–85.5 [65–89])	0.331
**Expired tidal volume; ml**	514 (511–532 [447–574])	517 (510–555 [462–573])	0.943
**Plateau pressure; cm H**_**2**_ **O**	14 (13–15 [12–19])	14 (13–14 [12–18])	0.486
**Static compliance; ml.cm H**_**2**_ **O** ^**-1**^	65.2 (59–70 [40.5–77.3])	60 (58.1–69.6 [38.8–77.7])	0.616
**PaCO**_**2**_ **; mmHg**	36.5 (33.9–37.7 [30–47.8])	36.1 (34.6–37.7 [32–43.8])	0.942
**PaO**_**2**_ **/F**_**I**_ **O**_**2**_ **; ratio**	402 (346–474 [300–571])	397 (319–455 [184–524])	0.734
**Cardiac index; l.min** ^**-1**^ **.kg** ^**-1**^	2.3 (2.2–2.5 [2–2.7])	2.4 (2.1–2.5 [1.9–3.5])	0.883
**Stroke volume variation; %**	6 (4–12 [2–12])	8 (6.3–9 [5–13])	0.261
**VD**_**phys**_ **/V**_**TE**_ **; %**	37 (34.2–39.2 [32.2–51.6])	37.7 (36–40 [30.3–41.9])	0.83
**VD**_**shunt**_ **/V**_**TE**_ **; %**	8.5 (7.5–9.3 [4.9–17.7])	11.1 (10.5–13 [4.2–19.6])	0.076
**VD**_**aw**_ **/V**_**TE**_ **; %**	13.3 (12.7–14.5 [10.7–23.9])	13.4 (10.8–16.4 [5.6–19.8])	0.65
**VD**_**alv**_ **/V**_**TE**_ **; %**	12.1 (11.3–15.6 [9.6–19.5])	12.4 (11–13.4 [9.5–16.7])	0.375

The initial ventilator settings used the same volume-controlled ventilation strategy for both groups at T_Baseline_. Values are reported as median (IQR [range]). *P*-values are from the Mann-Whitney test.

Abbreviations: VCV, volume-controlled ventilation; PC-IRV, pressure-controlled inverse ratio ventilation; FVC, forced vital capacity; FEV_1_, forced expiratory volume in 1 second; *P*aO_2_/F_i_O_2_, partial pressure of oxygen in arterial blood/fraction of inspiratory oxygen; VD_phys_, physiological dead space; V_TE_, expired tidal volume; VD_shunt_, shunt dead space; VD_aw_, airway dead space; VD_alv_, alveolar dead space.

During intervention, the I/E ratio was set at 2/1 for 10 patients and at 1.5/1 for 4 patients in the PC-IRV group. and at 1/2 for all 13 patients in the VCV group. Stroke volume variation in the PC-IRV group was higher compared with that in VCV group. The plateau pressure, P_a_CO_2_, and stroke volume variance increased with time, while the expired tidal volume and static compliance decreased with time.

The VD_phys_/V_TE_ and VD_shunt_/V_TE_ were decreased in PC-IRV group compared with those in VCV group, and both dead space rates increased with time in both groups. The VD_aw_/V_TE_ increased with time, but the difference between the groups was not significant. There were no significant changes in the VD_alv_/V_TE_ (Table 3).

**Table 3:** Respiratory and hemodynamic parameters during intervention.

Variable	Time period	VCV group (n = 13)	PC-IRV group (n = 14)	^a^*p*-value
**Respiratory rate; breaths.min** ^**-1**^	T_20min_	12 (12–14 [12–18])	12 (12–13.5 [12–14])	0.831
	T_2h_	12 (12–14 [12–18])	12 (12–14.8 [12–16])	0.957
^**b**^***p*-value**		0.174	0.055	
**Expired tidal volume; ml**	T_20min_	497 (456–510 [302–525])	480 (424–521 [391–576])	0.793
	T_2h_	489 (427–504 [308–517])	479 (410–514 [354–573])	0.43
^**b**^***p*-value**		0.005	0.194	
**Plateau pressure; cm H**_**2**_ **O**	T_20min_	24 (21–25 [18–29])	22 (20.3–24.8 [18–27])	0.525
	T_2h_	25 (22–26 [19–29])	23 (21.3–26 [19–29])	0.495
^**b**^***p*-value**		0.007	0.009	
**Static compliance; ml.cm H**_**2**_ **O** ^**-1**^	T_20min_	27.5 (23.6–32.5 [12.8–40.5])	29.1 (21.8–32.2 [17.3–44.1])	0.905
	T_2h_	24.9 (21–30.6 [13–37.2])	27.1 (19.8–28.4 [14.4–41.1])	0.793
^**b**^***p*-value**		<0.001	<0.001	
**PaCO**_**2**_ **; mmHg**	T_20min_	40.6 (38.5–43.8 [36.5–55])	39.4 (37.9–40.6 [35.4–43.5])	0.244
	T_2h_	45.1 (42–49.3 [37.7–60.7])	43.6 (40.1–48.3 [37.1–51.3])	0.332
^**b**^***p*-value**		0.005	<0.001	
**PaO**_**2**_ **/F**_**I**_ **O**_**2**_ **; ratio**	T_20min_	393 (316–470 [216–558])	381 (299–443 [171–500])	0.528
	T_2h_	396 (336–415 [173–560])	359 (302–439 [211–495])	0.72
^**b**^***p*-value**		0.328	1	
**Cardiac index; l.min** ^**-1**^ **.kg** ^**-1**^	T_20min_	2.6 (2.2–2.7 [1.9–4.1])	2.3 (2–3.1 [1.7–3.5])	0.543
	T_2h_	2.4 (2–2.7 [1.8–3.9])	2.2 (2–2.5 [1.8–3.4])	0.789
^**b**^***p*-value**		0.239	0.324	
**Stroke volume variation; %**	T_20min_	6 (4–9 [2–13])	9.5 (9–11 [8–16])	0.029
	T_2h_	9 (6–10 [4–14])	12.5 (10–13 [6–17])	0.02
^**b**^***p*-value**		0.014	0.114	
**VD**_**phys**_ **/V**_**TE**_ **; %**	T_20min_	33.4 (32.4–34.7 [25.2–56.4])	26.2 (24.5–28.1 [19.3–29.8])	<0.001
	T_2h_	38.9 (35.2–42.3 [30.8–59.8])	30.1 (26.9–33.7 [22.2–41.6])	<0.001
^**b**^***p*-value**		<0.001	0.002	
**VD**_**shunt**_ **/V**_**TE**_ **; %**	T_20min_	9.5 (4.5–10 [1.4–21])	2.2 (-0.2–6.3 [-1.4–8.9])	<0.001
	T_2h_	12.6 (8.9–16.4 [3.7–23.2])	4.9 (3–8.4 [-1.6–17.1])	0.003
^**b**^***p*-value**		0.001	0.03	
**VD**_**aw**_ **/V**_**TE**_ **; %**	T_20min_	10.1 (9.2–13.6 [7.9–26.2])	9.7 (9–11.2 [1.3–13.3])	0.519
	T_2h_	10.7 (9.8–14.5 [9.2–25.9])	10.4 (9.9–12.7 [7.9–15.3])	0.458
^**b**^***p*-value**		0.04	0.003	
**VD**_**alv**_ **/V**_**TE**_ **; %**	T_20min_	13.3 (12–15.3 [9.1–18.6])	13 (11.6–14.3 [9.4–21.5])	0.65
	T_2h_	13.7 (12.2–15.2 [10.8–18.7])	13.6 (12.9–14.2 [11.4–16.8])	1
^**b**^***p*-value**		0.305	0.391	

Values are presented as median (IQR [range]).

^a^*P*-values according to the group were obtained using the Mann-Whitney U test.

^b^*P*-values for the difference between T_20min_ and T_2h_ were obtained using Wilcoxon’s signed rank test.

Abbreviations: VCV, volume-controlled ventilation; PC-IRV, pressure-controlled inverse ratio ventilation; T_20min_, 20 min after intervention; T_2h_, 2 h after intervention; *P*aO_2_/F_i_O_2_, partial pressure of oxygen in arterial blood/fraction of inspiratory oxygen; VD_phys_, physiological dead space; V_TE_, expired tidal volume; VD_shunt_, shunt dead space; VD_aw_, airway dead space; VD_alv_, alveolar dead space.

**Fig 3:**
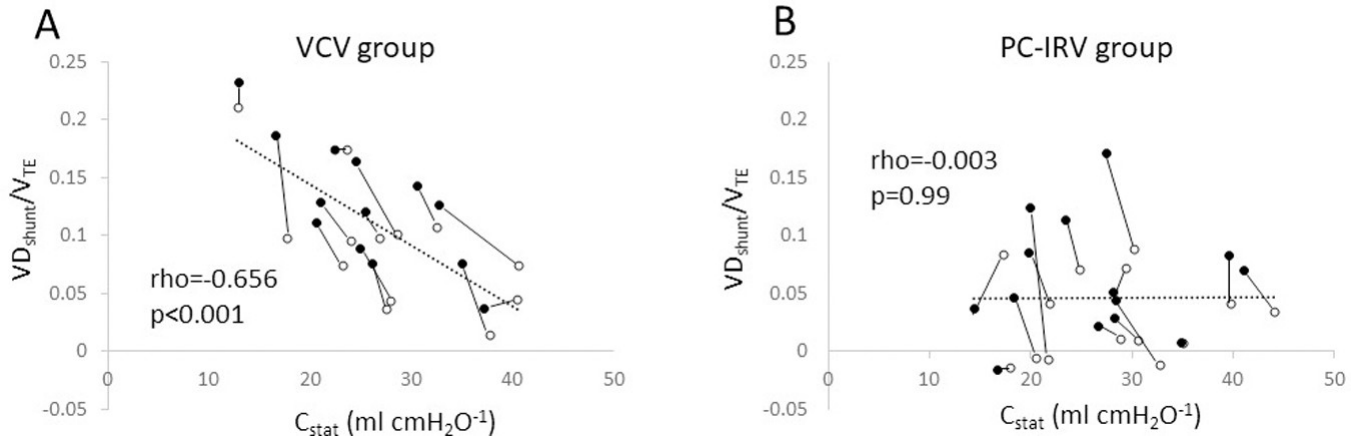
Correlation between static compliance and shunt dead space ratio (shunt dead space/expired tidal volume). The correlation between static compliance and shunt dead space ratio was evaluated using Spearman’s rank correlation at T_20min_ (open circle) and T_2h_ (closed circle) in the volume-controlled ventilation (A) and pressure-controlled inverse ratio ventilation (B) groups.

The VD_phys_/V_TE_ and VD_shunt_/V_TE_ were negatively correlated with static compliance in the VCV group (rho = -0.474; p = 0.015, rho = -0.656; p < 0.001, respectively), though not in the PC-IRV group (rho = -0.208; p = 0.285, rho = -0.003; p = 0.99, respectively) (Fig 3).

No instances of respiratory complications were recorded.

## Discussion

We observed that PC-IRV reduced the VD_phys_/V_TE_, suggesting that this ventilator mode improved the total of all ${\overset{\cdot }{V}}_{A}/\overset{\cdot }{Q}$ mismatches compared with VCV. However, the VD_phys_/V_TE_ increased with time and static compliance decreased with time in both groups. The reduction of static compliance with time may be an important finding to analyze the mechanism of changes in dead space induced by the time factor.

The VD_shunt_/V_TE_ is a functional evaluation of relative hyper-perfusion. The improvement in VD_shunt_/V_TE_ by PC-IRV indicates that prolonged plateau time encourages the expansion of slow-opening alveoli sufficiently and facilitates gas diffusion from the pulmonary artery to the alveoli, contributing to the improvement of intra-alveolar ${\overset{\cdot }{V}}_{A}/\overset{\cdot }{Q}$ <1 mismatch. The findings that the VD_shunt_/V_TE_ was increased with time in both the groups, suggest that atelectasis developed with time, leading to an increase in intra-alveolar ${\overset{\cdot }{V}}_{A}/\overset{\cdot }{Q}$ = 0 mismatch in both the groups. In general, the I:E ratio of 1:2, i.e., a long expiratory time, contributes to the development of atelectasis, and a BAP of 5−10 cm H_2_O is usually recommended to prevent atelectasis in laparoscopic surgery [13–16]. We observed that VD_shunt_/V_TE_ was negatively correlated with static compliance in the VCV group. It is likely that the observed positive-end expiratory pressure (PEEP) of 5 cm H_2_O used in the VCV group was insufficient in preventing the development of atelectasis with time in the Trendelenburg position with CO_2_ pneumoperitoneum accompanying very low respiratory compliance. In PC-IRV strategy, the I/E ratio was adjusted by observing the expiratory flow-time wave, which shortens the expiratory time in various situations and induces moderate total PEEP to prevent atelectasis. However, expiratory flow is not stable and is affected by respiratory compliance and flow resistance changes (asthma, sputum, atelectasis, muscle relaxation, patient posture, pneumoperitoneum pressure, surgical procedure, BAP). Thus, without BAP, establishing sufficient total PEEP in the PC-IRV group throughout the study period might have been difficult. We observed that VD_shunt_/V_TE_ was not correlated with static compliance in this group, suggesting that the influence of low static compliance in the development of atelectasis was smaller than that in the VCV group. Thus, sufficient total PEEP with a short expiratory time, PC-IRV with an I:E ratio of 1:1−1.5:1, and a BAP of 5−7 cm H_2_O might be a safe and practical strategy.

The VD_aw_/V_TE_, which is a functional evaluation of the airway space volume, did not show a significant improvement with PC-IRV. Our previous study has shown that PC-IRV reduces VD_aw_/V_TE_ significantly compared with VCV with a pause ratio of 0% (1). A pause ratio of 20% in the VCV strategy in this study would have diminished the differences. These results suggested that a prolonged plateau time enhanced gas diffusion from the alveoli to the airway, contributing to the improvement of extra-alveolar ${\overset{\cdot }{V}}_{A}/\overset{\cdot }{Q}$ = ∞ mismatch. However, this effect was small. Increase in VD_aw_/V_TE_ with time suggested that airway space volume increased with time in both groups, in accordance with increasing plateau pressure, contributing to an increase in extra-alveolar ${\overset{\cdot }{V}}_{A}/\overset{\cdot }{Q}$ = ∞ mismatch.

The VD_alv_/V_TE_ is a functional evaluation of relative hyperinflation. Similar to the lung recruitment maneuver, PC-IRV can increase the intrathoracic pressure and reduce venous return. These effects may be enhanced during hypovolemia [17]. Although a prolonged plateau time may enhance gas diffusion from the pulmonary artery to the alveoli, excessive plateau pressure or circulatory suppression might increase intra-alveolar ${\overset{\cdot }{V}}_{A}/\overset{\cdot }{Q}$ >1 mismatch. Thus, PC-IRV has the potential risk of increasing VD_alv_/V_TE_ in a high-plateau pressure condition with circulatory suppression. In this study, stroke volume variation was increased in PC-IRV. However, stroke volume variation and cardiac index were controlled within the normal range. The finding that there was no significant change in the VD_alv_/V_TE_ by PC-IRV indicates that PC-IRV was successfully managed with moderate plateau pressure and circulatory dynamics. There was no significant change in the VD_alv_/V_TE_ as a result of the time factor, representing no indication of pulmonary infarction in either group.

The conventional lung-protective or open-lung approach for ventilation consists of low tidal volume with moderate BAP [18–20]. However, these strategies seem to be based on VCV with an I:E ratio of 1:2, and there is no reason to choose it in case of low respiratory compliance with a muscle relaxant. Inflation of the alveoli with heterogeneous expansion disorder without hyperinflation of normal alveoli and preventing atelectasis would improve ${\overset{\cdot }{V}}_{A}/\overset{\cdot }{Q}$ mismatch, resulting in reduced physiological dead space. PC-IRV can be an alternative to open-lung approach for ventilation in cases of general anesthesia using muscle relaxants for robot-assisted laparoscopic radical prostatectomy or low respiratory compliance. However, it is neither commonly used nor safe. Furthermore, it needs an anesthesia ventilator equipped with expiratory flow-time wave monitor to avoid lung hyperinflation, adequate hemodynamic management, moderate muscle relaxant, and the skill and care of an anesthesiologist.

There are several limitations to our study. Firstly, neither auto-PEEP nor total PEEP were measured. It is desirable for PC-IRV to maintain a stable and sufficient total PEEP. Secondly, we did not use volumetric oxygraphy and the efficiency of O_2_ uptake was not evaluated. The effects of PC-IRV on oxygenation remain controversial [21–27]. The PaO_2_/FiO_2_ ratio was not significantly different between the VCV and PC-IRV groups, suggesting that the prolongation of plateau time did not improve the O_2_ uptake efficiency in patients with healthy lungs. The best ventilatory setting for O_2_ uptake efficiency may be different from that for the minimization of VD_phys_.

## Conclusions

Our study found that PC-IRV reduced the physiological dead space, suggesting an improvement in the total ${\overset{\cdot }{V}}_{A}/\overset{\cdot }{Q}$ mismatch due to the inflation of the alveoli affected by heterogeneous expansion disorder without hyperinflation of the normal alveoli. However, shunt dead space increased with time, suggesting that atelectasis developed with time in both the VCV and PC-IRV groups.

## Supporting information

S1 File Japanese protocol submitted to IRB. (DOCX)

S2 File English protocol. (DOCX)

S3 File CONSORT checklist. (DOCX)

S4 File Supplementary information of novel theory of volumetric capnography. (DOCX)

S5 File Dataset. (XLSX)
