# Supplementary material for: Changes in dead space components during pressure-controlled inverse ratio ventilation: A secondary analysis of a randomized trial
Source: PLoS One. 2021 Oct 13;16(10):e0258504. doi: 10.1371/journal.pone.0258504 (PMC8513857; doi:10.1371/journal.pone.0258504)
Supplement: S4 File — (DOCX) [file pone.0258504.s004.docx]

**Supplementary data**

**1. Components of dead space and volumetric capnography**

In traditional volumetric capnography, the expired CO_2_ fraction on the Y axis is plotted against the V_TE_ on the X axis. This analysis has been called the single breath test-CO_2_ (SBT-CO_2_). Normally, the expirogram is divided into three phases. Phase I represents CO_2_-free and pure dead space, phase II represents the transition between airway and alveolar gas, and phase III represents alveolar gas. Phase II ends at the crossing point of the slope of phase II and the slope of phase III. We present a volumetric capnogram with transpositioned x-y axis, as shown in Fig. 1. In 1948, Fowler applied the equal area method to phase II of the nitrogen expirogram after pure O_2_ inspiration, and he claimed that it was the geometric representation of Bohr’s formula.^14^ Thereafter, this dividing line was called the ‘Fowler Line’. In 1981, Fletcher and colleagues^15^ presented a review and a theoretical analysis of dead space by SBT-CO_2_, and they showed the geometric representation of Enghoff’s modification of Bohr’s formula. They drew a line of F_a_CO_2_ above the CO_2_ expirogram, and the area between this line and the CO_2_ expirogram was defined as physiological dead space (VD_phys_), and then airway dead space (VD_aw_) was defined by applying Fowler’s equal area method to phase II. Finally, the area between the F_a_CO_2_ line and Phase III of the CO_2_ expirogram was defined as alveolar dead space (VD_alv_). VD_phys_ and its subdivisions of VD_aw_ and VD_alv_ were shown as areas corresponding to each of the VDs on the SBT-CO_2_ expirogram. VD_aw_ separated by the Fowler Line is not a static volume of the respiratory tract but a dynamic and functional volume affected by various aspects of the flow pattern, such as flow rate, respiratory rate and I/E ratio.^15^ Fletcher and colleagues defined VD_alv_ as VD_phys_ - VD_aw_ geometrically. Regardless of the name ‘alveolar dead space’, the VD_alv_ was supposed to include the effect of venous admixture.^15^ In 2006, Tang, Turner and Baker proposed a new equal area method to represent VD_phys_ on the volumetric axis and made it easy to compare with other VDs.^21^ We will call this VD_phys_ dividing line the ‘Tang Line’ (Fig. 1).

参考：Fletcher1981


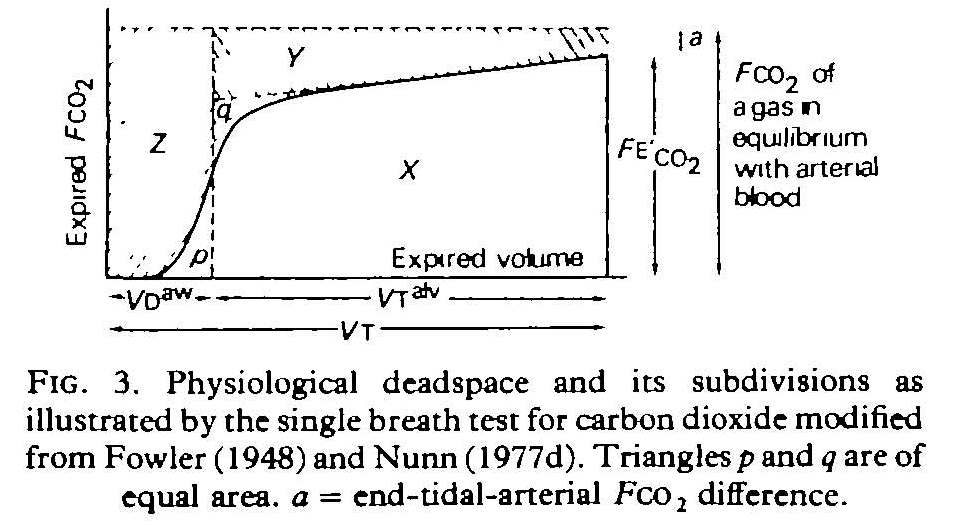


**2. Novel volumetric capnography and VD_resp_**

We used a non-invasive breath-by-breath volumetric capnography monitoring device developed by Senko Medical Instrument Co., Ltd., Tokyo, Japan, to estimate dead space components based on a novel analytic method. This device uses mainstream PHASEIN IRMA (Masimo Sweden AB), an infrared CO_2_ sensor for CO_2_ measurement, and EZ-Flow (TREYMED, Inc., US), a fixed orifice, differential pressure device for volume measurement. Analogue data output from these sensors are sent to the A/D converter, and the digital data are sent to a PC. Specialised software for novel dead space analysis was developed in Microsoft Visual Studio using the C language. The first unique point of this method is that the X axis represents FCO_2_ and the Y axis represents tidal volume for geometrical reasons. Second is that this method is based on a new theoretical model that can simultaneously determine the respiratory dead space (VD_resp_) and F_A_CO_2_ geometrically in a breath-by-breath manner, as shown in Fig. 3. The volume from the start of expiration to the mixed expired CO_2_ fraction point on the Y axis is defined as VD_resp_. Then, F_A_CO_2_ is defined as VCO_2_･(V_TE_ - VD_resp_)^-1^. Similar to VD_Bohr_, VD_resp_ is a functional evaluation of the difference of the CO_2_ partial pressure between alveolar and mixed expired gas [VD_resp_ = V_TE_･(P_A_CO_2_ - P_E_CO_2_)･P_A_CO_2_^-1^]. For further details, refer to Figs. 2 and 3 and their explanations.

**3. New dividing method of phase II and III**

VD_aw_ is analysed by Fowler’s equal area method non-invasively and geometrically too. The third unique point of this novel volumetric capnography is that the maximum point of multiplication of (x, y) values on the expirogram was used for the dividing method of phase II and III. To determine the VD_aw_ by applying Fowler’s equal area method to the second phase, it is essential to understand how to divide phase II and III properly and stably. The most popular method of determining the dividing line is by applying linear regression to each phase and dividing at the crossing point of their two lines. But this method has the contradiction of using linear regression without determination of the ranges of the two phases. As a new method that can eliminate this arbitrariness, we focused on the changes of the inner rectangular area that is inscribed in one of the corners to a point (x, y) on the expirogram. This area increases initially, reaches the maximum at a certain point (x, y), and decreases. Because both phases normally have different inclinations, such changes are necessary. Therefore, this method of dividing phase II and III at the maximum point of multiplication of (x, y) values on the expirogram is thought to be reasonable (Fig. 2).

**4. Validation of determination of dead space**

Prior to the clinical study, we performed a model lung CO_2_ mixing test to validate our methods to determine dead space. A 12-V DC fan was set inside the model lung (TTL, Michigan Instruments, USA) for gas mixing to simulate an alveolar unit. Extension tubes of dead space volume of 30/60/90/120/150 mL were added to the dead space volume (50 mL) of the mainstream CO_2_ sensor, flow sensor, heat and moisture exchanger, and Y-piece. Thus, the total dead space volume (VD_tot_) of the model lung varied from 80 to 200 mL in steps of 30 mL. An anaesthetic ventilator was set in VCV mode with TV 400 mL, RR 10 min^-1^, and I/E ratio 1/2. The CO_2_ flow rate (VCO_2_) into the model lung was controlled by a mass flow meter (Yamatake Corp., Tokyo, Japan) and varied from 100 to 200 mL min^-1^ in steps of 25 mL min^-1^. VCO_2_ RR^-1^ is the exhaled tidal volume of CO_2_, and it varied from 10 to 20 mL in steps of 2.5 mL. The area of the CO_2_ fraction-volume loop (VCO_2_) was analysed for each breath and compared with the above-mentioned VCO_2_ RR^-1^. VD_resp_ was analysed by the method described in Figs. 2 and 3 and compared with VD_tot_. According to the results of these model lung CO_2_ mixing tests, VCO_2_ was very close to the values of VCO_2_ RR^-1^ (VCO_2_ = 1.04・VCO_2_ RR^-1^, R^2^=0.994), and also, VD_resp_ was very close to VD_tot_ (VD_resp_ = 0.963・VD_tot_, R^2^ = 0.965) (Supplementary figure). The results of this experiment show the validity of VD_resp_ under conditions without VD_shunt_ and VD_alv_. In this model lung CO_2_ mixing test, simulation of VD_shunt_ and VD_alv_ is technically difficult. In vivo comparison of VD_resp_ and VD_Bohr_ would be required to validate VD_resp_ in the presence of VD_shunt_ and VD_alv_. This comparison appears practically impossible because no reliable method to determine VD_Bohr_ is available in clinical settings. Therefore, we suppose that there would be no experimental measure to completely prove the validity of our estimation of VD_Bohr_ with VD_resp_. However, we believe that our method to determine VD_resp_ as the substitute of VD_Bohr_ has a rational basis even though our results from this model experiment only provide incomplete support for the validity of our estimation.

**Supplementary figure** **legend**

Results of the model lung CO_2_ mixing test. To validate the accuracy of VCO_2_ as exhaled tidal volume of CO_2_ and respiratory dead space (VD_resp_) as dead space volume, a model lung CO_2_ mixing test was performed. Ventilator settings were VCV mode, TV 400 mL, RR 10 min^-1^, and I/E ratio 1/2. Number of breaths was 30 for each condition. CO_2_ flow rate (VCO_2_) into the model lung was controlled by a mass flow meter (Yamatake Corp., Tokyo, Japan) and varied from 100 to 200 mL/min in steps of 25 mL min^-1^ for VCO_2_ validation. VCO_2_ RR^-1^ is exhaled tidal volume of CO_2_, and it varied from 10 to 20 mL in steps of 2.5 mL. The area of the CO_2_ fraction-volume loop (VCO_2_) was analysed for each breath and compared with VCO_2_/RR (left panel). VCO_2_ was fixed at 150 mL min^-1^ for validation of VD_resp_. Extension tubes of 30/60/90/120/150 mL were added to the dead space (50 mL) of the CO_2_ and flow sensors, HME, and the Y-piece. VD_tot_ is the total dead space volume of the model lung. VD_resp_ was analysed by the method described in Figs. 2 and 3 and compared with total dead space (VD_tot_) (right panel).

**References**

21. Tang Y, Turner MJ, Baker AB. A new equal area method to calculate and represent physiologic, anatomical, and alveolar dead spaces. *Anaesthesiology* 2006; **104**:696–700.
